# Supplementary material for: Metagenomic Insights and Genomic Analysis of Phosphogypsum and Its Associated Plant Endophytic Microbiomes Reveals Valuable Actors for Waste Bioremediation
Source: Microorganisms. 2019 Sep 23;7(10):382. doi: 10.3390/microorganisms7100382 (PMC6843645; doi:10.3390/microorganisms7100382)
Supplement: Supplementary file 1 [file microorganisms-07-00382-s001.zip › Suppl. Materials/Supplementary Experimental.docx]

**Supplementary Experimental**

- 1. *Inductively coupled plasma optical emission spectrometry (ICP-OES) analysis of phosphogypsum*

The phosphogypsum was dried at 60 °C and was ground in fine powder. The elements were extracted in refluxing with 65% HNO_3_ (Suprapur^®^, Merck KGaA, Germany) for 48 h. Before analysis by ICP-OES, the dilution of samples (100 times) was performed by weighing.

The ICP-OES used for these analyses was an OPTIMA 2100 DV from Perkin Elmer, with AS-93 plus autosampler. The gases used were argon 5.0, nitrogen 5.0 and compresses air, with the following flow rate: for the plasma 15 mL/min, for auxiliary 0.2 mL/min, for nebulizer 0.65 mL/min.

Sample was injected in plasma at 1.50 mL/min (power 1300 Watts, axial mode and equilibration time 15 s) with three replicates of measure for each element.

The internal standard (IS) was an Yttrium solution in 2% HNO_3_ (Atomic spectroscopy standard, PerkinElmer Pure, 2% HNO_3_, 1000 ppm), concentration at 1 mg/mL. This IS was added to every sample to control the quality of the plasma.

The standard solutions for calibration were made in 2% HNO_3_ - MiliQ water (18 Ω), from PlasmaCAL-SCP33MS standard solution (SCP Science, Courtaboeuf, France).

Analysis was carried out on 24 elements:

| Symbol | Wave Length (nm) |
| --- | --- |
| Y (Internal Standard) | 371.029 |
| Cd | 228.802 |
| Pb | 220.354 |
| As | 193.697 |
| Hg | 253.652 |
| Co | 228.616 |
| Symbol | Wave Length (nm) |
| Ni | 231.604 |
| Cu | 327.395 |
| Al | 396.153 |
| Ba | 233.527 |
| Cr | 267.716 |
| Fe | 238.204 |
| Mn | 257.610 |
| Mo | 202.031 |
| Zn | 206.200 |
| Se | 196.026 |
| Rb | 780.023 |
| Sn | 189.927 |
| Sr | 460.733 |
| Li | 670.784 |
| Ag | 328.068 |
| Na | 589.592 |
| Mg | 285.213 |
| K | 766.490 |
| Ca | 371.933 |

The parameters of quantification were as follows.

| Peak Algorithm | Peak Area |
| --- | --- |
| Pts/peak | 7 |
| Overlap correction / Background corr. | None, 2-point |
| Peak Algorithm | Peak Area |
| Calibration | Lin, Calc Int. |
| Y (IS), check min – max (%) | 80 – 120% |

- 1. *Bacterial DNA extraction and 16S rRNA amplification*

High molecular weight genomic DNA suitable for either genome sequencing or 16S-rDNA amplification was isolated using the UltraClean^®^ Microbial DNA Isolation Kit (QIAGEN, Basel, Switzerland) according to manufacturer specifications. DNA integrity was ascertained by visual inspection of DNA after electrophoresis through 1.5% agarose gel analysis and quantity was estimated using Qubit Fluorometric Quantitation (Thermo Fisher, Switzerland). Genomic DNA was then used as template in 16S-rDNA PCR amplification using primers fD1 (5′-AGAGTTTGATCCTGGCTCAG-3′) and rP2 (5′-ACGGCTACCTTGTTACGACTT-3′) [1,2]. PCR amplifications were carried out in a final volume of 25 μL with 2.5 μL 10×Ex Taq buffer (20 mMTris–HCl, pH 8.0, 100 mM KCl), 2 μL 2.5 mM dNTP mixture,0.5 μM of fD1 and rP2, 1-unit Taq DNA polymerase (Promega, Dübendorf, Switzerland) and 5 ng genomic DNA using a Biometra T-one thermal cycler (Labgene, Chatel-Saint-Denis, Switzerland). Cycling conditions were as follows: an initial denaturation step at 95 °C for 1 min followed by 35 cycles (denaturation at 94 °C for 30 s, annealing at 55 °C for 30 s and extension at 72 °C for 20 s and final extension step at 72 °C for 5 min). Resulting PCR amplicons have then been purified using a Minelute PCR Purification Kit (Qiagen, Basel, Switzerland) following manufacturer’s recommendations.

- 1. *16S-rDNA sequencing and phylogenetic analysis*

Sequencing of 16S-rDNA purified PCR amplicons were performed on both directions using a BigDye^™^ Terminator v3.1 Cycle Sequencing Kit and primers fD1 and rP2 as described in Slama et al. [3]. Sequencing reactions were resolved using ABI 3130 XL facilities available at the iGE3 [Institute of Genetics and Genomics in Geneva, University of Geneva Medical Center (CMU), Switzerland]. Consensus sequences were generated by manual editing of raw sequence files using SeqMan^TM^II (DNASTAR, Madison, WI, United States). Blasting of the consensus sequences against NCBI’s GenBank sequence database allowed recovery of their closest relatives needed for subsequent phylogenetic placement of the sequences. Phylogenetic analysis of phosphogypsum and four plants endophytic bacterial microbiomes along with their closest relatives helped ascertain their exact phylogenetic position. Briefly, alignments of bacterial isolates and their closest relatives generated using the multiple sequence alignment web-based program MAFFT [4] were used to generate phylogenetic trees based on the Neighbor-joining (NJ) algorithm [5] MEGA v.6 [6] with computed evolutionary distances using the Kimura 2-parameter [7]. Bootstrap resampling support of the data sets with 1000 replications allowed the validation of the branches in the different phylogenetic trees.

- 1. *Measurement of PGP activities of S. fruticosa, S. mollis, M. nodiflorum and A. indicum endophytic communities*
     1. *Phosphate solubilization*

Phosphate solubilization assay was measured according to the protocol described by Slama et al. [3]. Briefly endophytic communities were inoculated into Pikovskaya’s agar plates and incubated at 30 °C for minimum 4 days. Colonies with a clear halo around them were considered as phosphate solubilizers.

Pikovskya’s agar medium was prepared as follows: in (g/L) yeast extract (0.5), dextrose (10), (NH4)_2_SO_4_ (0.5), KCl (0.2), MgSO_4_ (0.1), MnSO_4_ (0.0001), FeSO_4_ (0.0001), agar-agar (20) supplemented with 5 g/L of an insoluble phosphate (tri-calcium phosphate Ca_3_(PO_4_)_2_). Then, 0.003% of Rose Bengal was added to the medium as an indicator.

- - 1. *Nitrogen fixation*

*Suaeda fruticosa*, *S. mollis*, *M. nodiflorum* and *A. indicum* endophytic communities were subjected to nitrogen fixation assay. Briefly, endophytes were inoculated onto nitrogen free bromothymol blue (NFB) medium. After incubation of plates at 30 °C for 48 h, the appearance of yellow color around colonies indicates their ability to fix nitrogen.

NFB medium was prepared as follows: in (g/L) Malic acid (5), KOH_4_.K_2_HPO_4_ (0.5), FeSO_4_.7H2O (0.05), MnSO_4_. H_2_O (0.01), MgSO_4_.7H_2_O (0.1), NaCl (0.02), CaCl_2_.2H_2_O (0.01), Na_2_MoO_4_.2H_2_O (0.002), Agar 15 g. The pH was adjusted to 6.8 with KOH. Afterwards, 2 mL of bromothymol blue was added (0.5 % alcoholic solution) to NFB medium as an indicator.

- - 1. *HCN production*

HCN production assay was investigated on nutrient agar medium supplemented with 4.4 g/L glycine. Following inoculation of endophytes, a sterile Whatman filter paper no. 1 was flooded with solution containing 0.5 % picric acid in 2 % sodium carbonate and then placed in the upper lid of the Petri plates. The plates were then sealed with parafilm and incubated at 30°C for 7 days. HCN producer endophytes were identified by the apparition of an orange-reddish color on the Whatman filter paper.

- - 1. *ACC deaminase activity*

The endophytic isolates were inoculated on DF salts minimal medium containing (g/L) 4 g KH_2_PO_4_, 6 g Na_2_HPO_4_, 0.2 g MgSO_4_.7H_2_O, 2 g glucose, 2 g gluconic acid and 2 g citric acid, 18 g Agar. A solution of trace elements was added to DF salts medium containing: 1mg FeSO_4_.7H_2_O,10µg H_3_BO_3_, 11.19 µg MnSO_4_.H_2_O, 124.6 µg ZnSO_4_.7H_2_O, 78.22 µg CuSO_4_.5H_2_O, and 10 µg MoO_3_. After autoclaving DF salts medium, ACC solution filter-sterilized through a 0.2-mm membrane (30 µmol/plate) was added to DF medium as sole source of nitrogen.

- - 1. *Siderophore Production*

Chrome azurol S Agar medium was used for siderophore production assay. The ability of *S. fruticosa*, *S. mollis*, *M. nodiflorum* and *A. indicum* endophytic communities to produce siderophore was expressed by the appearance of yellow-orange color around colonies.

Chrome Azurol S medium (CAS) was prepared as a mixture of 60.5 mg/50 mL Chrome Azurol S in distilled water (DW), 72.9 mg/40 mL CTAB in DW, 10 mL of 1 mM FeCl_3_.6H_2_O prepared in 10 mM HCl. Then, the mixture was added to 900 mL of King B (42.23 g/L) to obtain 1L of CAS medium.

- 1. *Screening of phosphogypsum and S. fruticosa, S. mollis, M. nodiflosum and A. indicum endophytic bacterial microbiomes for:*
     1. *Antibiotic resistance*

The disc diffusion method was performed to assess the resistance of the phosphogypsum and plant associated bacterial and endophytic isolates to different antibiotics belonging to different classes. Briefly, bacterial cultures were streaked on Mueller-Hinton Agar medium and five antibiotic discs of ampicillin (50 µg/mL), penicillin G (50 µg/mL), streptomycin (50 µg/mL), tetracycline (15 µg/mL), and rifamycin (50 µg/mL) were placed on the surface of the plates. After bacterial growth for 48 hours at 37 °C the inhibition zone around the antibiotic disc was measured according to Ma et al. [8]. A minimum of three assays have been conducted and representative results collected.

- - 1. *Metal resistance*

Phosphogypsum and plant associated bacterial and endophytic isolates were tested for their abilities to tolerate different metals including Magnesium (MgSO_4_), Cadmium (CdCl_2_), Aluminum (AlCl_3_) Iron (FeCl_3_), Nickel (NiCl_2_), Zinc (ZnSO_4_), Mercury (HgCl_2_), Copper (CuCl_2_), Lead (PbNO_3_) and Vanadium (NH_4_VO_3_) following the protocol described by Khan et al. [9]. Briefly, the isolates were inoculated on LB agar media supplemented with 100 ppm of each metal. The inoculated plates were incubated at 37 °C for 48 h. All experiments were conducted in triplicate.

- - 1. *Salinity (NaCl) tolerance*

Salinity tolerance of the phosphogypsum and plant associated bacterial and endophytic isolates was tested following the protocol of Slama et al. [3]. Briefly, the isolates were inoculated on LB agar media supplemented with different concentrations of NaCl ranging from 1 to 4. The inoculated plates were incubated at 37 °C for 48 h. All experiments were conducted in triplicate and representative results of bacterial growth recorded.

- - 1. *Pesticides and effluent degradation*

For pesticides, the phosphogypsum and plant associated bacterial and endophytic isolates were inoculated on minimum medium containing NaCl (2.25 g), KCl (0.105 g) CaCl_2_(0.12 g), NaHCO_3_ (0.05 g) agar (15 g) and amended with 1000 mg/L of the insecticides (dimethoate, imidacloprid and permethrin) and the herbicide (Glyphosate). Plates were incubated for 48 h at 37 °C and the diameter of the growth halo around colonies were measured and expressed in mm. All experiments were carried out at least in triplicate.

For effluents, PG, petroleum and textile wastewater effluents were added to minimum medium at different concentrations 20, 40 and 60 g/L. Then, the phosphogypsum and plant associated bacterial and endophytic isolates were inoculated on the above described media and the plates incubated for 48 h at 37 °C. The results were expressed in mm by measuring the halo of growth diameter. Results of at least three replicates were recorded.

- 1. *Bacterial genome sequencing, assembly and annotation*

Five genomes of the phosphogypsum bacterial isolates described in this study (PG 1, PG 9, PG 17, PG 18 and PG 26, Table S1) were processed according to Slama et al. [3] using facilities available at the iGE3 genomics platform of the University of Geneva (https://www.ige3.unige.ch/). Briefly, sequencing libraries were generated from DNA of the isolates using Illumina’s TruSeq sample preparation reagents according to manufacturer recommendations. MiSeq sequencing starting material were then inserted into the MiSeq reagent cartridge and loaded on the instrument along with the flow cell. After low quality reads filtering genome assembly is attempted on remaining reads. Bacterial genome sequences (PG 1, PG 9, PG 17, PG 18 and PG 26) were deposited in GenBank and received the following accession numbers SDFO00000000, SDFP00000000, SDFQ00000000, SDFR00000000 and SDFS00000000 respectively.

- 1. *Selection and phylogenomic analysis of phosphogypsum bacterial isolates*

Publicly available *B. albus* isolates genomes that have more than 98% 16S-rRNA gene sequence similarity with the type strain N35-10-2T, were downloaded from GenBank (Table S1). The completeness and contamination rates of the collected *B. albus* genomes were assessed using the CheckM program v.1.0.9 [10]. All genomes displayed ≥90.0% completeness and ≤10.0% contamination suggesting their high quality and justifying their use in the study subsequent analysis. Genome to Genome Distance (GGD) and Average Nucleotide Identity (ANI) values allowed selection of genomes phylogenomically belonging to the species *B. albus*. GGD and ANI values were estimated using the server-based genome-to-genome distance calculator (v. 2.13) and the ANI online server according to Meier-Kolthoff et al. [11] and Yoon et al. [12], respectively. species and sub-species cut-off for GGD and ANI analysis were those suggested by default analysis (70% and 95–96%, respectively). The reference sequence alignment-based phylogeny builder (REALPHY) [13] was used to generate whole genome alignments. That were subsequently used by the Bayesian inference (BI), as implemented in MrBayes v. 3.2.1 [14]. The best evolutionary model for each data partition was obtained using the software MrModelTest v. 2.3 [15]. The heating parameter was set at 0.15 and the Markov Chain Monte Carlo analysis of four chains was started in parallel from a random tree topology and lasted until the average standard deviation of split frequencies came below 0.01. Trees were saved each 100 000 000 generations, and the first 25 % of saved trees were discarded as the ‘burn-in’ phase and posterior probabilities determined from the remaining trees. The resulting phylogenetic tree was printed with Geneious v. 5.6.7 [16].

- 1. *DNA extraction from phosphogypsum and S. fruticosa, S. mollis, M. nodiflorum and A. indicum plant materials and metagenomic analysis*

Genomic DNA was extracted from phosphogypsum using a PowerSoil DNA Isolation Kit (MoBio) according to manufacturer recommendations (Ozyme, Saint Quentin En Yvelines, France). For DNA isolation from plant material, plant tissues were thoroughly washed under running tap water and surface sterilized as previously described in Mefteh et al. [17]. Briefly, plant materials were sliced in pieces and treated by incubation into 75% ethanol and the 0.5% sodium hypochlorite for 2 min. Plant materials were then washed with sterile distilled water for at least six times. Efficacy of sterilization was ascertained by plating an aliquot of the last wash on TSA media and no bacterial growth was observed. Additionally, imprinting plant pieces on TSA media revealed no bacterial growth. DNA from plant tissues was isolated using PowerPlant^®^ Pro DNA Isolation Kit (Ozyme, Saint Quentin En Yvelines, France). DNA yield and its integrity were checked using protocols described in Cherrad et al. [18]. Ribosomal RNA from DNA samples were amplified using 16S-rDNA bacterial specific primers (B969F and BA1406R) amplifying the V6-V8 region of the small subunit rRNA gene [19]. For all samples, a unique DNA barcode was introduced to the PCR amplicons by the forward PCR primers. Barcodes used for Illumina MiSeq sequencing were 10 bp in length.

PCR amplifications were performed using the GoTaq^®^ Flexi DNA Polymerase (Promega, Dübendorf, Switzerland) in a final volume of 30 μL containing 20-50 ng of template DNA, 5 μL of 5X buffer, 1μL of dNTP at 40 μM each, 1μL of each tagged primer at 10 μM and 0.5 μl of Taq Polymerase at 5 Unit/μL. Following an initial denaturation step at 95 °C for 3 min, PCR was cycled 30 times at 94 °C for 30 s, 57 °C for 1 min, 72 °C for 1 min, and a final extension at 72 °C for 10 min. Negative controls were run on each PCR. Successful amplification of the predicted amplicon size was confirmed by gel electrophoresis.

Each Polymerase chain reaction product was quantified using a Qubit Fluorometer (Invitrogen) and then all samples were pooled into equimolar concentrations (0.50 µg) and purified using the Wizard^®^ SV Gel and PCR Clean-Up System (Promega, Dübendorf, Switzerland).

Pooled samples were used for library preparation using the Illumina paired-end kit, cluster generation and 300 bp paired-end sequencing on the Illumina MiSeq sequencer. Image processing, base calling and error estimation were processed using CASAVA 1.8.2 (Illumina, San Diego, USA).

The merged 16S sequences were subjected to quality filtering [20]. Then chimera have been excluded by screening against the GOLD database [21]. Mothur v.1.41.1 [22] and QIIME v.1.8 [23] as implemented in Nephele (<https://nephele.niaid.nih.gov/>) were used to analyse the data.

- 1. *Isolation of S. fruticosa, S. mollis, M. nodiflorum A. indicum endophytic bacterial microbiome*

For isolation of culturable endophytes, *S. fruticosa*, *S. mollis*, *M. nodiflorum*, *A. indicum* plant materials were processed immediately after their collection. A total of 25 leaves and roots of each plant species were thoroughly washed under tap water. They were cut in small pieces (about 5×5mm) and then surface sterilized. Pieces of leaves and roots were then placed in petri dishes containing Tryptic Soy Agar (TSA) media. After plate incubation at 25 °C, they were checked daily and bacteria growing out of the plant tissue transferred to fresh TSA media. Similar to studies in the field, effectiveness of surface sterilization was ascertained by making imprints of disinfected plant fragments on TSA plates and no bacterium growth revealed for the four plant tissues [3,17,24,25]. Identification of bacterial isolates was performed based on morphological features prior to molecular analysis. The isolates were stored in the culture collection of NextBiotech, Tunisia.

- 1. *Bacterial isolates growth conditions*

Unless indicated all bacterial and endophytic isolates have been grown on LB agar media. For liquid cultures bacterial and endophytic isolates have been grown in liquid LB medium at 37 °C overnight with a shaking speed of 180 rpm.

**References**

[1] W.G. Weisburg, S.M. Barns, D.A. Pelletier, D.J. Lane, 16S ribosomal DNA amplification for phylogenetic study, J. Bacteriol. 173 (1991) 697–703.

[2] N. Mlaik, J. Bouzid, I. Ben Hassan, S. Woodward, L. Belbahri, T. Mechichi, Unhairing wastewater treatment by *Bacillus pumilus* and *Bacillus cereus*. Desalin. Water Treat. 54 (2015) 683–689.

[3] H.B. Slama, H. Cherif-Silini, A. Chenari Bouket, M. Qader, A. Silini, B. Yahiaoui, F.N. Alenezi, L. Luptakova, M.A. Triki, A. Vallat, T. Oszako, M.E. Rateb, L. Belbahri, Screening for Fusarium antagonistic bacteria from contrasting niches designated the endophyte Bacillus halotolerans as plant warden against Fusarium, Front. Microbiol. 9 (2019) 3236. https://doi.org/10.3389/fmicb.2018.03236.

[4] K. Katoh, H. Toh, Recent developments in the MAFFT multiple sequence alignment program, Brief. Bioinf. 9 (2008) 286–298. https://doi.org/10.1093/bib/bbn013.

[5] N. Saitou, M. Nei, The neighbor-joining method: a new method for reconstructing phylogenetic trees, Mol. Biol. Evol. 4 (1987) 406–425. https://doi.org/10.1093/oxfordjournals.molbev.a040454.

[6] K. Tamura, G. Stecher, D. Peterson, A. Filipski, S. Kumar, MEGA 6: Molecular Evolutionary Genetics Analysis Version 6.0, Mol. Biol. Evol. 30 (2013) 2725–2729. https://doi.org/10.1093/molbev/mst197.

[7] M. Kimura, A simple method for estimating evolutionary rates of base substitutions through comparative studies of nucleotide sequences, J. Mol. Evol. 16 (1980) 111–120.

[8] Y. Ma, M. Rajkumar, C. Zhang, H. Freitas, Beneficial role of bacterial endophytes in heavy metal phytoremediation, J. Environ. Manage. 174 (2016) 14–25. https://doi.org/10.1016/j.jenvman.2016.02.047.

[9] A.R. Khan, I. Ullah, A.L. Khan, G.S. Park, M. Waqas, S.J. Hong, B.K. Jung, Y. Kwak, I.J. Lee, J.H. Shin, Improvement in phytoremediation potential of Solanum nigrum under cadmium contamination through endophytic-assisted Serratia sp. RSC-14 inoculation, Environ. Sci. Pollut. Res. Int. 22 (2015) 14032–14042. https://doi.org/10.1007/s11356-015-4647-8.

[10] D.H. Parks, M. Imelfort, C.T. Skennerton, P. Hugenholtz, G.W. Tyson, CheckM: assessing the quality of microbial genomes recovered from isolates, single cells, and metagenomes, Genome Res. 25 (2015) 1043–1055. https://doi.org/10.1101/gr.186072.114.

[11] J.P. Meier-Kolthoff, A.F. Auch, H.P. Klenk, M. Goker, Genome sequence-based species delimitation with confidence intervals and improved distance functions, BMC Bioinformatics. 14 (2013) 60. https://doi.org/10.1186/1471-2105-14-60.

[12] S.H. Yoon, S.M. Ha, S. Kwon, J. Lim, Y. Kim, H. Seo, et al, Introducing EzBioCloud: a taxonomically united database of 16S rRNA and whole genome assemblies, Int. J. Syst. Evol. Microbiol. 67 (2017) 1613–1617. https://doi.org/10.1099/ijsem.0.001755.

[13] F. Bertels, O.K. Silander, M. Pachkov, P.B. Rainey, E. van Nimwegen, Automated reconstruction of whole-genome phylogenies from short-sequence reads, Mol. Biol. Evol. 31 (2014) 1077–1088. https://doi.org/10.1093/molbev/msu088.

[14] F. Ronquist, J.P. Huelsenbeck, MrBayes 3: Bayesian phylogenetic inference under mixed models, Bioinformatics. 19 (2003) 1572–1574. https://doi.org/10.1093/bioinformatics/btg180.

[15] J.A.A. Nylander, MrModeltest v.2.0 program distributed by the author. Sweden: Evolutionary Biology Centre, Uppsala University, 2004.

[16] A. Drummond, B. Ashton, S. Buxton, M. Cheung, A. Cooper, C. Duran, M. Field, J. Heled, M. Kearse, et al, Geneious v 5.6. <http://www.geneious.com>, 2012 (accessed 10 January 2019).

[17] F.B. Mefteh, A. Daoud, A. Chenari Bouket, F.N. Alenezi, L. Luptakova, M.E. Rateb, A. Kadri, N. Gharsallah, L. Belbahri, Fungal root microbiome from healthy and brittle leaf diseased date palm trees (Phoenix dactylifera L.) reveals a hidden untapped arsenal of antibacterial and broad spectrum antifungal secondary metabolites, Front. Microbiol. 8 (2017) 307. https://doi.org/10.3389/fmicb.2017.00307.

[18] S. Cherrad, A. Charnay, C. Hernandez, H. Steva, L. Belbahri, S. Vacher, Emergence of boscalid-resistant strains of Erysiphe necator in French vineyards, Microbiol. Res. 216 (2018) 79–84. https://doi.org/10.1016/j.micres.2018.08.007.

[19] A.M. Comeau, W.K. Li, J.E. Tremblay, E.C. Carmack, C. Lovejoy, Arctic Ocean microbial community structure before and after the 2007 record sea ice minimum. PLoS ONE. 6 (2011) e27492. https://doi.org/10.1371/journal.pone.0027492.

[20] R. Schmieder, R. Edwards, Quality control and pre-processing of metagenomic datasets, Bioinformatics. 27 (2011) 863–864. https://doi.org/10.1093/bioinformatics/btr026.

[21] T.B.K. Reddy, A.D. Thomas, D. Stamatis, J. Bertsch, M. Isbandi, J. Jansson, et al, The Genomes OnLine Database (GOLD) v. 5: a metadata management system based on a four level (meta)genome project classification, Nucleic Acids Res. 43 (2015) D1099–106. https://doi.org/10.1093/nar/gku950.

[22] P.D. Schloss, S.L. Westcott, T. Ryabin, et al, Introducing Mothur: Open-source, platform-independent, community-supported software for describing and comparing microbial communities, Appl. Environ. Microbiol. 75 (2009) 7537–7541. https://doi.org/10.1128/AEM.01541-09.

[23] J.G. Caporaso, J. Kuczynski, J. Stombaugh, K. Bittinger, F.D. Bushman, E.K. Costello, et al, QIIME allows analysis of high-throughput community sequencing data, Nat. Methods. 7 (2010) 335–336. https://doi.org/10.1038/nmeth.f.303.

[24] W. Sun, Z. Xiong, L. Chu, W. Li, M.A. Soares, J.F. Jr White, H. Li, Bacterial communities of three plant species from Pb-Zn contaminated sites and plant-growth promotional benefits of endophytic Microbacterium sp. (strain BXGe71), J. Hazard. Mater. S0304-3894(18)30079-7 (2018). https://doi.org/10.1016/j.jhazmat.2018.02.003.

[25] R. Lumactud, R.R. Fulthorpe, Endophytic bacterial community structure and function of herbaceous plants from petroleum hydrocarbon contaminated and non-contaminated sites, Front. Microbiol. 9 (2018) 1926. https://doi.org/10.3389/fmicb.2018.01926.
