# Supplementary material for: Metagenomic Insights and Genomic Analysis of Phosphogypsum and Its Associated Plant Endophytic Microbiomes Reveals Valuable Actors for Waste Bioremediation
Source: Microorganisms. 2019 Sep 23;7(10):382. doi: 10.3390/microorganisms7100382 (PMC6843645; doi:10.3390/microorganisms7100382)
Supplement: Supplementary file 1 [file microorganisms-07-00382-s001.zip › Suppl. Materials/Table S1.docx]

Table S1. List and description of the *Bacillus albus* isolates used in the study.

| **Species** | **Strain** | **Genome size (Mb)** | **GC%** | **Description** | **GenBank Accession** |
| --- | --- | --- | --- | --- | --- |
| ***Bacillus albus*** | [PFYN01](https://www.ncbi.nlm.nih.gov/genome/71466?genome_assembly_id=432725) | 4.93958 | 35.30 | Isolated from Silt in the tailings reservoir area, [China (Panzhihua](https://www.ncbi.nlm.nih.gov/biosample?term=%22geo_loc_name=China:Panzhihua%22%5battr%5d)) | [CP034548](https://www.ncbi.nlm.nih.gov/nuccore/CP034548.1) |
|  | [N35-10-2](https://www.ncbi.nlm.nih.gov/genome/71466?genome_assembly_id=394478) | 5.80624 | 35.00 | Isolated from sediments, [China (South China Sea](https://www.ncbi.nlm.nih.gov/biosample?term=%22geo_loc_name=China:%20South%20China%20Sea%22%5battr%5d)) | [MAOE00000000](https://www.ncbi.nlm.nih.gov/nuccore/MAOE00000000.1) |
|  | [PG 26](https://www.ncbi.nlm.nih.gov/genome/71466?genome_assembly_id=442457) | 5.89447 | 35.00 | Isolated from phosphogypsum piles, Tunisia | [SDFS00000000](https://www.ncbi.nlm.nih.gov/nuccore/SDFS00000000.1) |
|  | [PG 9](https://www.ncbi.nlm.nih.gov/genome/71466?genome_assembly_id=442458) | 5.89566 | 35.10 | Isolated from phosphogypsum piles, Tunisia | [SDFP00000000](https://www.ncbi.nlm.nih.gov/nuccore/SDFP00000000.1) |
|  | [PG 1](https://www.ncbi.nlm.nih.gov/genome/71466?genome_assembly_id=442459) | 5.89351 | 35.00 | Isolated from phosphogypsum piles, Tunisia | [SDFO00000000](https://www.ncbi.nlm.nih.gov/nuccore/SDFO00000000.1) |
|  | [PG 17](https://www.ncbi.nlm.nih.gov/genome/71466?genome_assembly_id=442460) | 5.89581 | 35.10 | Isolated from phosphogypsum piles, Tunisia | [SDFQ00000000](https://www.ncbi.nlm.nih.gov/nuccore/SDFQ00000000.1) |
|  | [PG 18](https://www.ncbi.nlm.nih.gov/genome/71466?genome_assembly_id=442558) | 5.89532 | 35.10 | Isolated from phosphogypsum piles, Tunisia | [SDFR00000000](https://www.ncbi.nlm.nih.gov/nuccore/SDFR00000000.1) |
