# Supplementary material for: Metagenomic Insights and Genomic Analysis of Phosphogypsum and Its Associated Plant Endophytic Microbiomes Reveals Valuable Actors for Waste Bioremediation
Source: Microorganisms. 2019 Sep 23;7(10):382. doi: 10.3390/microorganisms7100382 (PMC6843645; doi:10.3390/microorganisms7100382)
Supplement: Supplementary file 1 [file microorganisms-07-00382-s001.zip › Suppl. Materials/Table S2.docx]

Table S2. ICP-OES for Phosphogypsum waste.

| Elements | Phosphogypsum (mg/kg) ‘Sfax region’ |
| --- | --- |
| Cd | 17.177 |
| Pb | 1.955 |
| As | 1.815 |
| Hg | 0 |
| Co | 0 |
| Ni | 6.005 |
| Cu | 6.144 |
| Al | 790.107 |
| Ba | 14.803 |
| Cr | 37.286 |
| Fe | 1103.425 |
| Mn | 5.446 |
| Mo | 0 |
| Zn | 135.876 |
| Se | 9.217 |
| Rb | 2.514 |
| Sn | 0 |
| Sr | 163.470 |
| Li | 1.815 |
| Ag | 0 |
| Na | 5435.389 |
| Mg | 490.411 |
| K | 313.318 |
| Ca | 3664.460 |

Dry soil weight = 56.9 mg
