# Supplementary figures and images for: Metagenomic Insights and Genomic Analysis of Phosphogypsum and Its Associated Plant Endophytic Microbiomes Reveals Valuable Actors for Waste Bioremediation
Source: Microorganisms. 2019 Sep 23;7(10):382. doi: 10.3390/microorganisms7100382 (PMC6843645; doi:10.3390/microorganisms7100382)

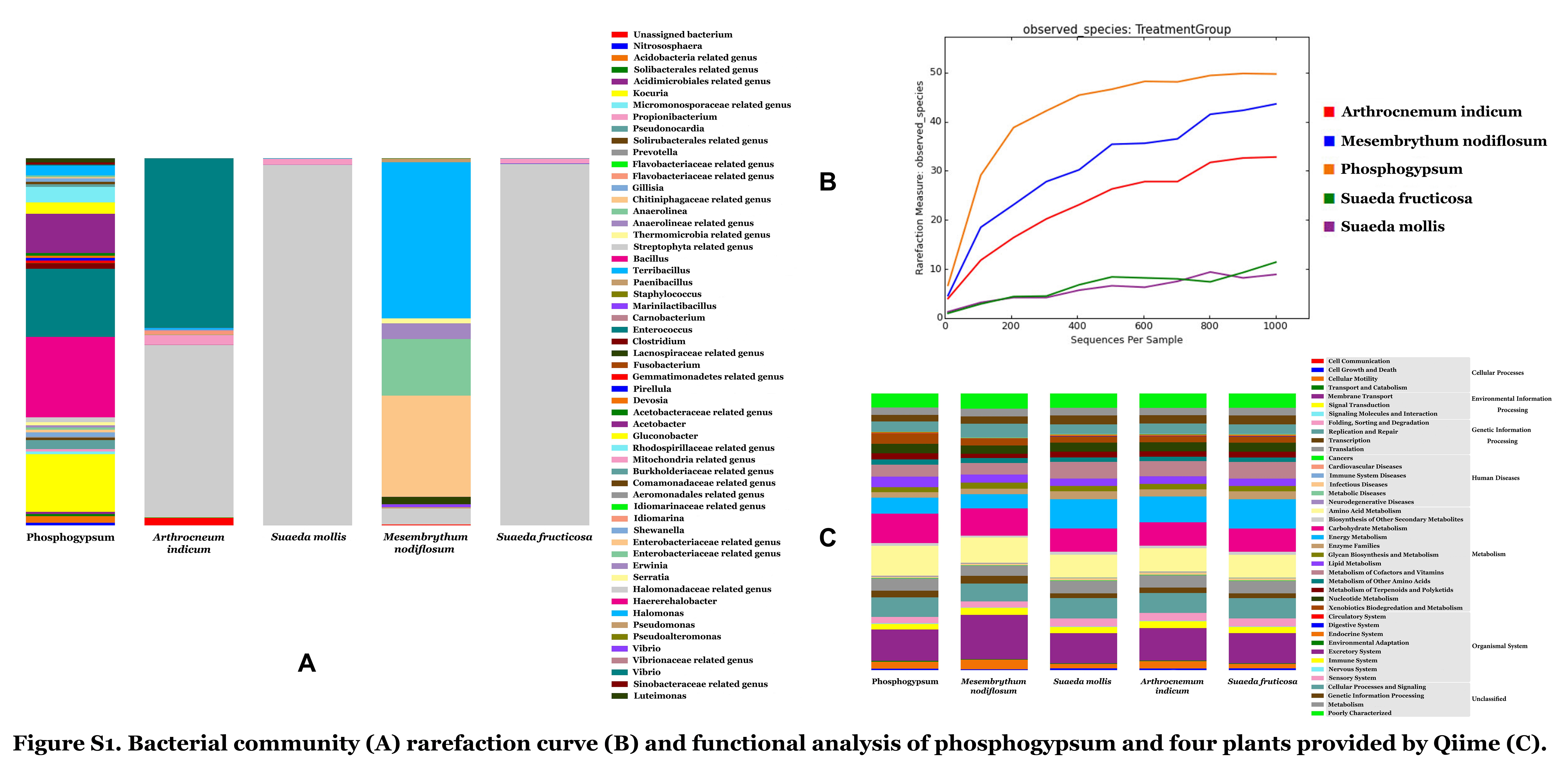

Supplement: Supplementary file 1 [file microorganisms-07-00382-s001.zip › Suppl. Materials/Figure S1.jpg]

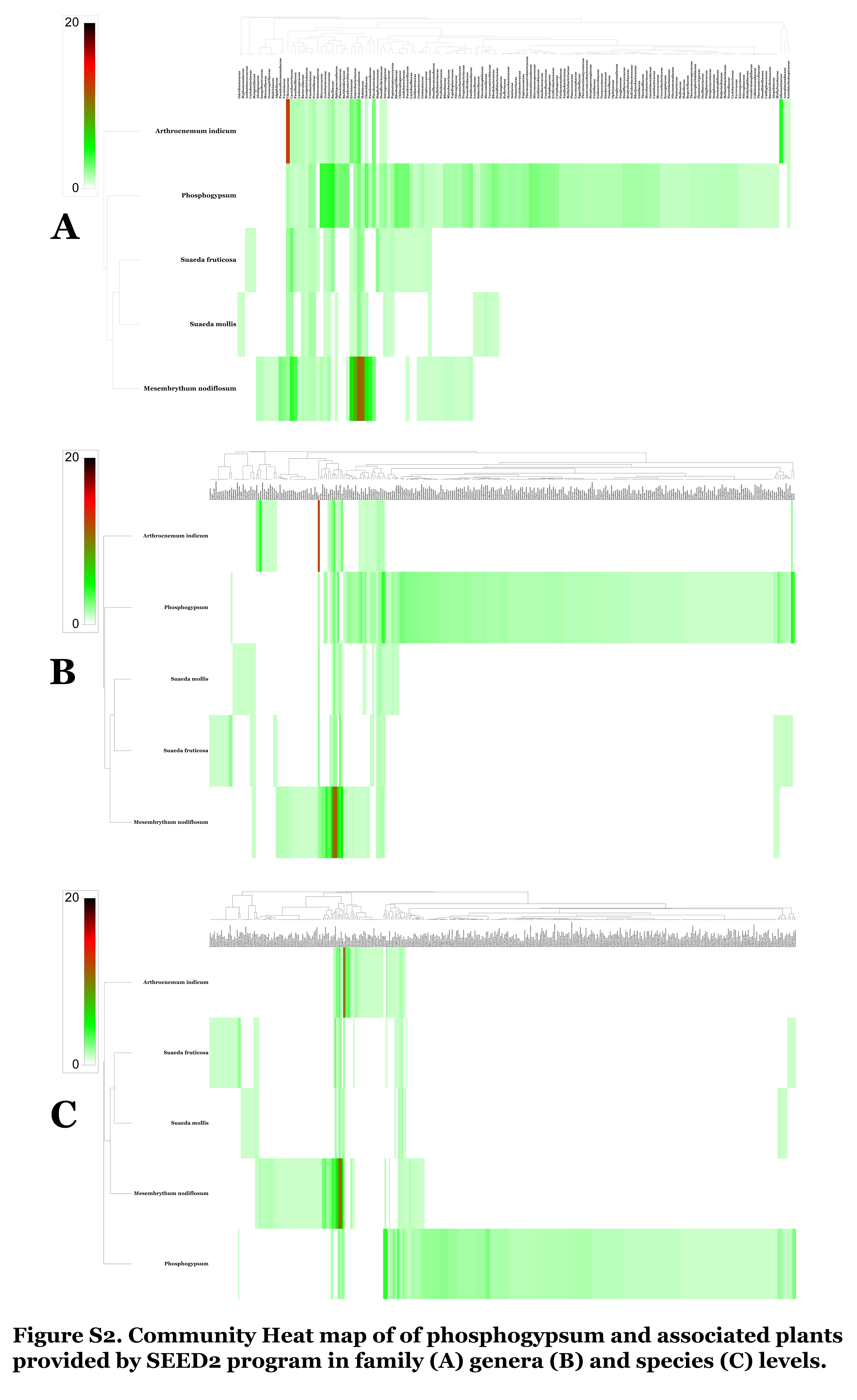

Supplement: Supplementary file 1 [file microorganisms-07-00382-s001.zip › Suppl. Materials/Figure S2.jpg]

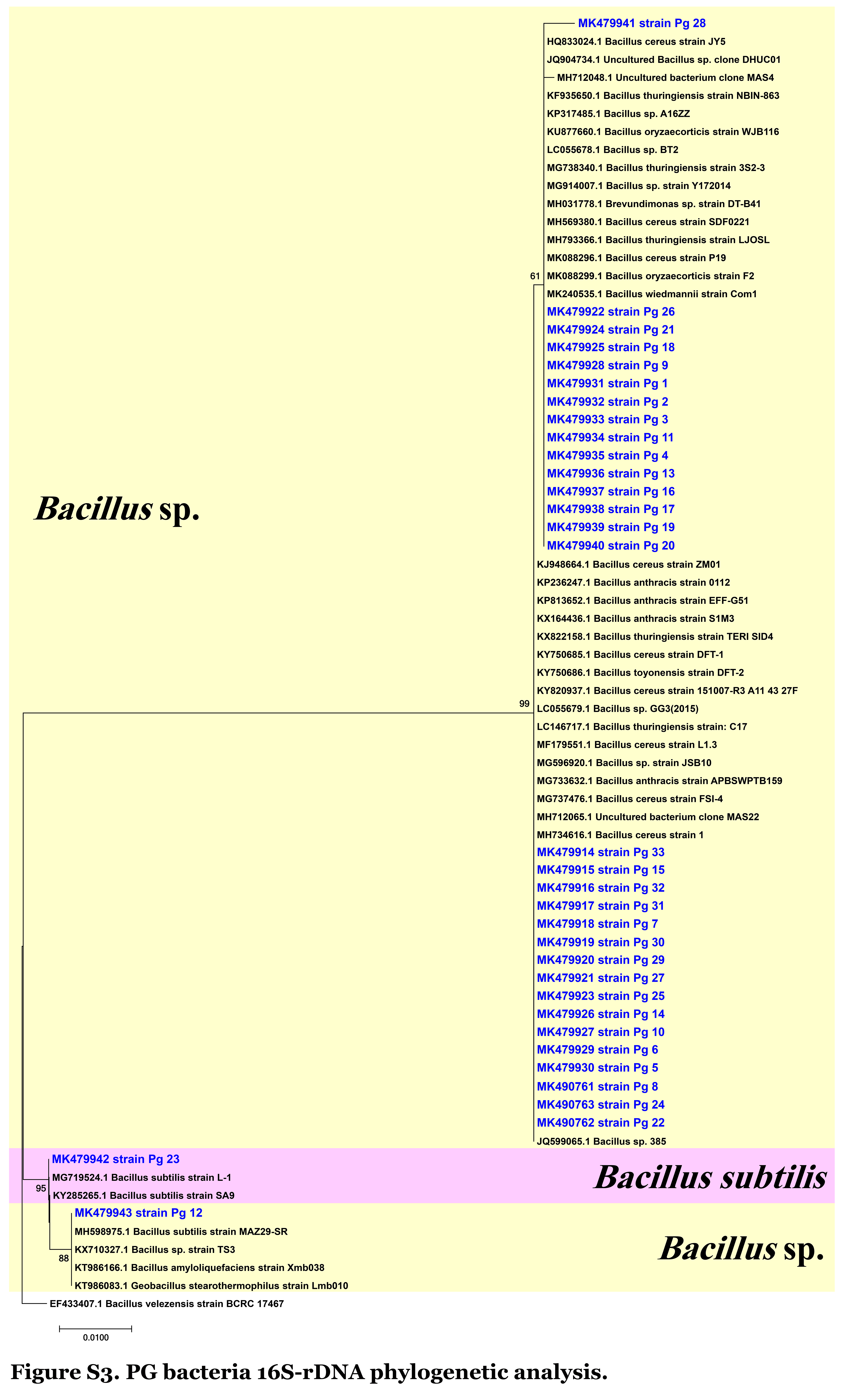

Supplement: Supplementary file 1 [file microorganisms-07-00382-s001.zip › Suppl. Materials/Figure S3.jpg]
